# Mesenchymal stromal cell and bone marrow concentrate therapies for musculoskeletal indications: a concise review of current literature

**DOI:** 10.1007/s11033-020-05428-0

**Published:** 2020-05-25

**Authors:** Christian Eder, Katharina Schmidt-Bleek, Sven Geissler, F. Andrea Sass, Tazio Maleitzke, Matthias Pumberger, Carsten Perka, Georg N. Duda, Tobias Winkler

**Affiliations:** 1 Center for Musculoskeletal Surgery, Charité - Universitaetsmedizin Berlin, Chariteplatz 1, 10117 Berlin, Germany; 2 Julius Wolff Institute, Charité - Universitaetsmedizin Berlin, Augustenburger Platz 1, 13353 Berlin, Germany; 3 Berlin Institute of Health Center for Regenerative Therapies, Charité – Universitaetsmedizin Berlin, Augustenburger Platz 1, 13353 Berlin, Germany; 4 Berlin-Brandenburg School for Regenerative Therapies, Charité – Universitaetsmedizin Berlin, Augustenburger Platz 1, 13353 Berlin, Germany

**Keywords:** Mesenchymal stromal cells, Orthopedic surgery

## Abstract

The interest on applying mesenchymal stromal cells (MSCs) in orthopedic disorders has risen tremendously in the last years due to scientific successes in preclinical in vitro and animal model studies. In a wide range of diseases and injuries of the musculoskeletal system, MSCs are currently under evaluation, but so far have found access to clinical use only in few cases. The current assignment is to translate the acquired knowledge into clinical practice. Therefore, this review aims at presenting a synopsis of the up-to-date status of the use of MSCs and MSC related cell products in musculoskeletal indications. Clinical studies were included, whereas preclinical and animal study data not have been considered. Most studies published so far investigate the final outcome applying bone marrow derived MSCs. In fewer trials the use of adipose tissue derived MSCs and allogenic MSCs was investigated in different applications. Although the reported results are equivocal in the current literature, the vast majority of the studies shows a benefit of MSC based therapies depending on the cell sources and the indication in clinical use. In summary, the clinical use of MSCs in patients in orthopedic indications has been found to be safe. Standardized protocols and clear definitions of the mechanisms of action and the mode and timing of application as well as further coordinated research efforts will be necessary for finally adding MSC based therapies in standard operating procedures and guidelines for the clinicians treating orthopedic disorders.

## Introduction

The entities being treated in orthopedics and trauma surgery range from circumscribed lesions such as tendon ruptures and meniscus tears to complex degenerative disorders (e.g. osteoarthritis, OA) and combinations of major trauma in polytraumatized patients. This variety demands a great need for specific therapeutic options and even with today’s high medical standards there remain conditions, where current gold standard treatments fail.

As example, core decompression in patients suffering from osteonecrosis of the femoral head is a well known and common practice to prevent patients from joint replacement operations. Larger lesion size and laterally located lesions are negative predictive factors for the minimally invasive treatment of femoral head osteonecrosis [1]. However, not only patients with risk factors fail applying this current gold standard treatment. New pathways to cure orthopedic diseases or prevent disease progression are highly needed.

The translation-oriented research on the biology of stem cells has risen continuously in the last decades, including the improvement of their isolation, expansion and further characterization as well as their clinical application [2, 3].

The cell types examined within this paper comply with the minimal criteria to define the mesenchymal stromal cell (MSC) that have earlier been labeled as mesenchymal stem cells [4].

MSCs were primarily experimentally characterized by Goujon in 1868 transplanting bone marrow (BM) in animal models and showing an osteogenic differentiation potential [5]. It is now generally accepted, that the largest quantity of MSCs can be found in the BM, but can also be isolated from adipose tissue, peripheral blood, placenta, and plenty of other sources [6, 7].

They have the potential to differentiate into fat, bone and cartilage tissue as well as, under specific conditions, into neuronal cells, hepatocytes, myocytes and others [6–8].

Following the guidance of the International Society for Cellular Therapy MSCs have to fulfill the following minimum criteria:

(I) plastic-adherent (standard culture conditions),
(II) must express CD105, CD73 and CD90,
(III) lack expression of CD45, CD34, CD14 or CD11b, CD79alpha or CD19 and HLA-DR surface molecules,
(IV) differentiate to osteoblasts, chondroblasts and adipocytes in vitro [9].

In 2019 Viswanathan et al. published their statement paper to clarify the different nomenclature of mesenchymal stromal cells versus mesenchymal stem cells (both abbreviated MSC). The study group commented on the above stated criteria for defining MSCs as criteria for in vitro expanded MSC. Especially the expression of surface markers CD34 and HLA-DR molecules in vivo is more complex than formerly expected. So, exemplarily, it was shown that in vivo MSCs are partly CD34 positive and the expression of CD34 increases when adding Insulin-like growth factor 1 in culture medium. The HLA-DR surface markers were shown to be influenced by the interaction with interferon gamma. Furthermore, they emphasize the need of clarifying the tissue of origin for the MSC isolation because of some apparent different characteristics. Additionally, a more functional definition of MSCs versus mesenchymal stem cells was recommended due to the lack of adequate surface markers to distinguish the cell populations. The term “mesenchymal stromal cells” describes a heterogenous cell population including fibroblasts, myofibroblasts and stem cells but excludes hematopoietic and endothelial cells. Therefore, further characterization of the MSC population via their functional profile additionally to phenotyping and the comparison with appropriate references (e.g. MSCs in rest) was recommended. In conclusion, the Viswanathan et al. emphasized the clear distinction of stem and stromal cells on basis of their functional abilities and characteristics to be kept. Only if tri-lineage differentiation potential is proofed in vivo and in vitro, the term “mesenchymal stem cells” has to be used [10].

The mechanism of action of MSCs include the capability of self-renewal and differentiation as well as their secretion of trophic factors and immunomodulatory effects, the latter having been identified as main mechanisms of action in most studies [11].

The other cellular product discussed within this paper is the so called BM aspirate concentrate (BMAC). In contrast to the MSCs as supplementary therapy, BMAC does not only include the stromal cell fraction with its higher concentrated number than in unconcentrated marrow but also a variety of different cell types and lineages. Exemplarily, the study group of Gangji et al. described, that after concentration of 400 ml of BMA from the iliac crest to a mean volume of 51 ± 1.8 ml, the reinserted concentrate contained 2.0 ± 0.3 × 10^9^ leukocytes and 92 ± 9/10^7^ fibroblast colony-forming units. The mononuclear cells within their cell product contained about 29% lymphoid cells, 4% monocytoid cells and 6% myeloid cells [12].

Following the tremendously growing data from basic research, MSCs have been translated to a wide range of clinical applications, but many are expected to follow in the next years. A sound assessment of evidence for their efficacy in each application and the quality of basic and translational research efforts are critical before recommending therapies to patients.

This review presents a concise overview of the current use of MSCs in orthopedic and orthopedic trauma surgery applications. The up-to-date status of therapies, which have reached clinical study level is discussed, excluding pre-clinical and animal studies without clinical translation so far. We also excluded systemic disorders such as osteoporosis, Osteogenesis imperfecta and hypophosphatasia and refer the reader to respective review articles, case series and clinical trials in this field [13–15].

The aim of this review is to critically evaluate the actual knowledge and potential possibilities of MSC use in orthopedic disorders (bone fractures, defects and non-unions/osteonecrosis/intervertebral disc degeneration, spinal cord injury, spinal fusion/muscle injury/tendon, ligament and meniscus injuries and degeneration/osteochondral defects/OA) for clinicians and researchers and to provide a basis for judgement on available and future therapeutic approaches with MSCs in the orthopedic and orthopedic trauma surgery field.

## Material and methods

A systematic research was performed using the PubMed database, Cochrane Library and the Web of Science database.

Inclusion criteria were

- application of MSCs (either as stand-alone treatment or in combination with other procedures),
- application of concentrated BMA (BMAC),
- clinical trials.

Exclusion criteria were the missing availability of full text publication, other languages than English and German, not-fitting topic, animal model data, pre-clinical studies, use of agents other than MSC-based (e.g. unprocessed BMA) and doublings of studies because of intersections of term searches.

Additionally, systematic reviews and literature reviews were included, to examine further studies within this topic.

**Fig. 1:**
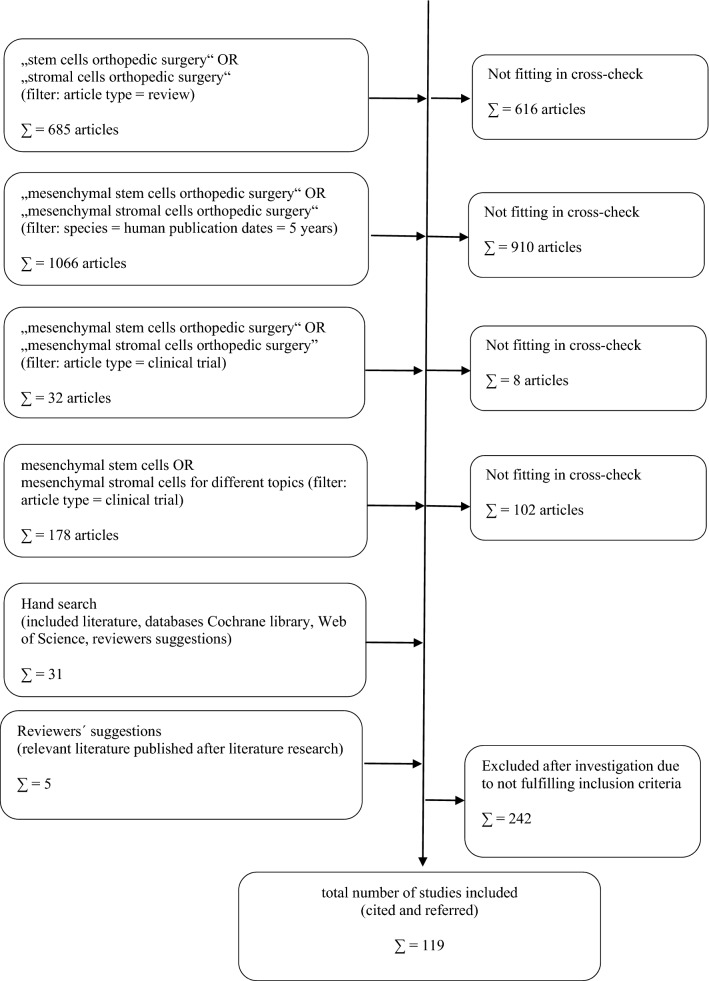
Literature research and analysis (“not fitting in cross check”: other diseases than orthopedic, pre-clinical studies, animal model data, not MSC cells used, use of not distinctly characterized cell products/ “inclusion criteria”: application of MSCs or BMAC, clinical trials)

The literature research was conducted as a four-step model (Fig. 1).

Therefore, primarily, the terms “stem cells orthopedic surgery” OR “stromal cells orthopedic surgery” were used with the filter “review”. From 685 studies displayed, 89 were chosen for further evaluation. The others did not fit in the topic after considering title and/or abstract (mainly because they analyzed other diseases than orthopedic disorders).

Secondary, the terms “mesenchymal stem cells orthopedic surgery” OR “mesenchymal stromal cells orthopedic surgery”, subgrouped for species = humans and publication dates = 5 years were analyzed. 1066 Studies were displayed and examined at first by title and abstract. A total of 176 were chosen for further evaluation according to our previously mentioned standards.

The third step included the search for “mesenchymal stem cells orthopedic surgery” OR “mesenchymal stromal cells orthopedic surgery”, subgrouped for article types = clinical trial. 32 Studies were displayed and 24 were included for the review article.

Finally, after identifying relevant topics and headlines for further investigation within this paper, a differentiated search for the following terms was performed (all filtered for article types = clinical trial):

1. “bone defect mesenchymal stem cells” OR “bone defect mesenchymal stromal cells” (31 results, 6 for further check),
2. “cartilage mesenchymal stem cells” OR “cartilage mesenchymal stromal cells” (33 results, 22 for further check),
3. “osteoarthritis mesenchymal stem cells” OR “osteoarthritis mesenchymal stromal cells” (23 results, 18 for further check),
4. “muscle mesenchymal stem cells” OR “muscle mesenchymal stromal cells” (40 results, 6 for further check),
5. “tendon mesenchymal stem cells” OR “tendon mesenchymal stromal cells” (11 results, 2 for further check),
6. “ligament mesenchymal stem cells” OR “ligament mesenchymal stromal cells” (4 results, 0 for further check),
7. “meniscus mesenchymal stem cells” OR “meniscus mesenchymal stromal cells” (2 results, 2 for further check),
8. “osteonecrosis mesenchymal stem cells” OR “osteonecrosis mesenchymal stromal cells” (10 results, 6 for further check),
9. “spine mesenchymal stem cells” OR “spine mesenchymal stromal cells” (17 results, 9 for further check),
10. “disc mesenchymal stem cells” OR “disc mesenchymal stromal cells” (7 results, 5 for further check).

Concluding, a hand search was done with regard to the references of cited and referred literature to identify further studies, missing after the literature scouting in the databases Cochrane Library, Web of Science.

After reviewing all the literature primarily included and marked “for further check” and applying the inclusion and exclusion criteria as well as after including special remarks from the reviewers with special hint to relevant literature published after performing the literature research for this article, a total of 119 studies were included in this review.

All studies were analyzed and described in relevant detail to give an overview of study design and results. Topics under certain headlines were summarized to help the reader identify relevant key points.

MSC-using and BMAC-using studies were included, but separated within the paragraph “results”.

## Results

**Fig. 2:**
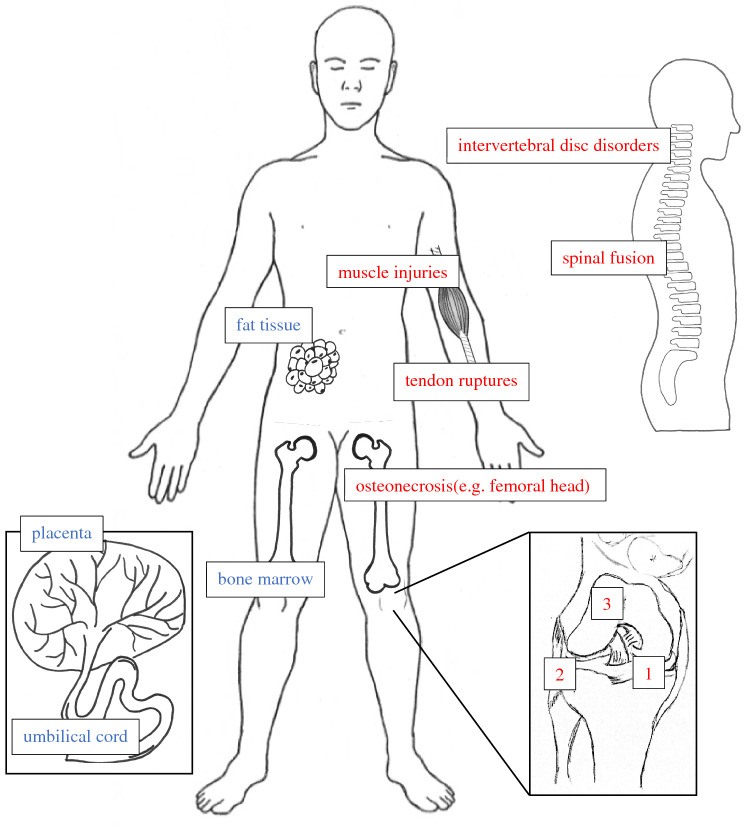
Sources (blue) and fields of application (red) for MSCs in orthopedic conditions, based on currently published clinical trials. 1 = Meniscus degeneration/damages, 2 = ligament ruptures/degeneration (here exemplarily collateral ligaments of the knee joint), 3 = articular surface/articular cartilage tissue degeneration or traumatic lesions. (Color figure online)

Figure 2 summarizes the potential sources and fields of application for MSCs discussed in this review.

Below, relevant clinical trials using MSCs in orthopedic disorders in patients are analyzed, described and summarized under specific headlines and topics. Case series are reported in addition when applications were considered fitting.

### Bone fractures, defects and non-unions

Larger bone defects and non-unions or delayed unions after insufficient healing of fractures or due to high-energy trauma, shot or blast injuries, infections, hereditary diseases or tumors remain a challenge in orthopedic surgery. MSCs can contribute to healing due to their potential of differentiation and due to secretion of specific factors, influencing the human immune system as well as their capability of interacting with other cells in vivo [16].

#### Studies using MSCs

**Šponer et al.** presented their findings in patients with aseptic loosening of total hip arthroplasty and accompanying bony defects. They performed a phase IIa clinical trial with two subgroups: the trial group (nine patients) received an absorbable sponge (ultraporous beta-tricalcium phosphate, Vitoss, Stryker®) serving as carrying medium for autologous BM derived stromal cells (BM-MSCs) (adherence selected, 15 ± 4.5 × 10^6^ BM-MSCs) whereas the control group (9 patients) received the carrier without cells into the femoral defect area. The group published the results with a minimum follow-up of 12 months [parameters: Harris Hip Score (HHS), pain score, radiographs, DEXA scan]. No serious adverse events (SAEs) were reported.

The results showed a comparable improvement of the HHS and pain scores in treatment and control groups. The radiologic evaluation showed significant differences: in the trial group two patients with cortical defects developed cortical repair and all of the nine patients showed trabecular remodeling in the filled medullary cavity.

In contrast, only one filled medullary cavity in the control group showed trabecular remodeling, four patients showed trabecular incorporation and the other four patients stayed without any changes; one patient with a cortical defect showed cortical healing as well. Radiolucency decreased in the trial group whereas it increased in the control group. The DEXA scan showed no statistical differences in both subgroups [17].

**Liebergall et al.** randomized 24 patients (n = 12 control group, n = 12 intervention group) in a prospective study to investigate the efficacy of MSC application in fractures of the distal third of the tibia (extraarticular) to prevent non-unions.

All patients underwent surgery with intramedullary nailing (21 cases) or percutaneous plate osteosynthesis (3 cases). The intervention group furthermore received an injection of a composite graft consisting of demineralized bone matrix (DBM), platelet-rich plasma (PRP) and sorted BM MSCs (CD105+ magnetic cell sorting, minimum of 5 × 10^6^ autologous BM-MSCs per sample) percutaneously into the fracture site.

In the follow-up examinations 12 months after surgery, all patients showed fracture healing on plain radiographs (three patients in the control group showed a delayed union at the 3 months post surgery follow-up). The time to union was significantly reduced from 3 to 1.5 months in the intervention group [18].

**Giannotti et al.** performed a case series with eight patients suffering from nonunions in the upper extremity. BM-MSCs were harvested from the iliac crest and further cultivated. MSCs were implanted in blood clots (autologous plasma gel and CaCl_2_) into the bony lesions additionally to a plate and screw osteosynthesis. The group reported no adverse events (AEs) (follow-up of 50.3 months) and stated bony healing in all cases (median of 6 months, range from 3.5 to 10 months) and a full regain of normal function [19, 20].

The group of **Dufrane et al.** published a case series in 2015 including six patients suffering from either musculoskeletal cancer diseases (osteosarcoma, Ewing sarcoma) or pseudarthrosis (congenital, acquired due to erythroblastopenia). They designed a three-dimensional graft using autologous DBM seeded with adipose-tissue derived MSCs (AD-MSCs) (enzymatically isolated and expanded). These 3D grafts were implanted into the defect sites. The group reported no acute side effects, but one case of bacterial infection requiring the removal of implants after 10 months. Three patients developed bony consolidation, whereas the other three patients showed no or only insufficient bony healing. Two of the three patients without sufficient bony healing suffered from congenital pseudarthrosis and one patient from osteosarcoma [21].

**Marcacci et al.** published their results of a clinical investigation on long bone repair using bioceramics with BM-MSCs in 2007. They examined 4 patients with large bone defects (4 cm tibia, 4 cm forearm, 7 cm distal humerus and 6 cm ulna). During the operative procedures macroporous cylinders (3 times a hydroxyapatite cylinder with a pore diameter of 614 ± 93 µm, 1 time an Engipore-ceramic and pore diameter of 431 ± 52 µm) seeded with MSCs were implanted into the bone defect. MSCs had been harvested from iliac crest aspirations, selected via plastic adherence and cultured for approximately 3 weeks.

No AEs were described. A callus formation as signal for beginning bony fusion was seen after 1 to 2 months after the operative procedures. The implant to bone consolidation was described as completed after 5–24 months post surgery. A regain of function of the treated extremity was described for all patients. In the follow-up (6 to 7 years after surgery), the group stated a completed integration of the implanted material [22].

**Quarto et al.** reported of three cases of substantial bone loss (4 to 7 cm, due to unsuccessful bone lengthening or traumatic loss). Bone defects were located at the tibia, ulna and humerus. The authors implanted a macroporous hydroxyapatite scaffold (in shape reflecting the bone defect) seeded with BM derived (BMD) osteoprogenitor cells (expanded ex vivo) with additional external fixation. The group reported osteointegration of the scaffold and full limb recovery (follow-up 15 to 27 months). No adverse advents were described during the investigation [23].

**Bajada et al.** reported of one case of tibial shaft non-union after high-velocity trauma which was treated with BM-MSC (adherence tested) seeded onto synthetic calcium sulphate pellets (4.8 mm and 3 mm). The pellets were impacted by hand into the defect site and covered with periosteum layer. After 8 weeks of the initial operation, bony healing was described and symptom reduction was obtained; after 2 years of follow-up treatment, a regain of normal function was reported [24].

*Conclusion* so far, the autologous bone graft is the gold standard for treating larger bone defects or non-unions after fractures, whereas further options—mainly biomaterial-based—are developed, examined and tested nowadays [25, 26]. The here listed and described clinical trials and case reports indicate that MSCs could be a future treatment option to further enhance bone healing in difficult cases and therefore improve patient’s functional outcomes.

The main problem that can be identified based on the existing literature is the insufficient failure and efficacy analysis of treated cases also based on the inhomogeneity of the groups and the absence of biomarker analyses. Furthermore, different combinations of cell products and scaffolds and different biomaterials used make it hard to judge on the effect of the cells and the influence of the biomaterial because of the few studies directly comparing control and treatment groups. Furthermore, the majority of all patients has been investigated in case reports or series and not in prospective controlled trials. Large bone defects are always a combined problem of substance loss, vascularity, scar healing preventing regrowth of original bone tissue and secondary problems such as infections. This makes this indication one of high medical need but not of easy addressability. Approaches with combinational products using cells in defined scaffolds always increase the complexity of the experiment and are therefore more prone to fail than one component tests. This might also have contributed to the decrease in numbers of publications in the field. We are now slowly gaining more insight into scaffold biology and it can be expected that biological large bone reconstruction will gain traction again in the future [25].

### Osteonecrosis

Osteonecrosis is caused by a local reduction or disturbance of blood supply. The reasons for this condition range from hereditary vascular malformations to microangiopathies and rheological changes in the human body [27].

Most of the clinical investigations are dealing with the local osteonecrosis of the femoral head (ONFH), which therefore deliver the most reliable and comparable data. Other forms of osteonecrosis are examined exemplarily.

#### Studies using MSCs

In 2012, the group of **Zhao et al.** published a randomized controlled trial (RCT) including 100 patients (104 ONFH hips) subdivided into 2 groups: 51 (44 completed follow-up) hips treated with core decompression alone and 53 hips with core decompression and implantation of autologous BM-MSCs (adherence selected).

The authors did not report any AEs. 10 of 44 hips treated in the control group showed radiological progression with the need for conversion to THA in 5 cases. In contrast, 2 of the 53 hips treated with MSC application showed progression with no need for THA. Additionally, the intervention group had a greater improvement in HHS than the core decompression group. The volumetric measurement of the osteonecrotic areas showed a significant decrease in the MSC group compared to the control group [28]. However, no information has been given concerning blinding in this work.

The same group published an uncontrolled case series in 2015 evaluating 24 patients with 31 affected hips (ONFH). They implanted a tantalum rod with BM-MSCs and described an improvement in HHS but the need for conversion to THA in five hips. Radiographic progression was observed in three cases, others showed no disease progression [29].

**Aoyama et al.** published their findings of 10 patients with ONFH treated with autologous BM-MSCs (differentiation potential and cytogenetics were analyzed before application) with beta tricalcium phosphate granules serving as scaffold (in combination with vascularized iliac bone grafts), and core decompression. The bone volume increased and the JOA (Japanese Orthopedic Association) score improved as well, except in two cases where clinical progress occurred [30].

**Chen et al.** presented an uncontrolled case series of nine patients suffering from ONFH. They conducted a study with a clinical and radiological follow-up of 24 months after infusion of allogeneic human umbilical cord-derived MSCs into the right femoral artery. A decrease of the necrotic areal in MRI as well as an increase in clinical HHS up to 12 months post-intervention, yet with a consecutive decrease after 24 months was reported [31].

#### Studies using BMAC

**Rastogi et al.** reported on a randomized trial including 40 patients with 60 ONFHs with a mean follow-up of 24 months. Two groups were analyzed (both received an intralesional injection):

- *group 1* (*intervention*) treated with isolated BM mononuclear cells (isolation via Ficoll density separation),
- *group 2* (*control*) treated with unprocessed BMA.

Patients were examined clinically (via HHS) and radiologically (plain radiographs and MRI). The grading of disease progression was done according to the ARCO staging system. No AEs or complications were reported. The HHS in both groups increased with no statistical significance between both groups.

The radiological examination for the intervention group showed an improvement in stage I and II hips; the stage III hips stayed without changes. In the control group, none of the stage I hips showed any changes, but in four cases (stage II and III hips) a deterioration was noticed.

Three of these cases needed further surgery (conversion to total hip arthroplasty).

The group concluded the isolated BM mononuclear cells to be beneficial compared to unprocessed BMA in early stages of ONFH [32].

**Tabatabaee et al.** performed a randomized trial with 18 patients (28 cases of ONFH) subdivided into 2 groups: core decompression alone versus core decompression plus injection of BMAC into the necrotic lesion. The surgeon and operating room staff were unblinded, physicians involved in follow-up were blinded for the patient’s individual treatment. The group stated a reduction in VAS and WOMAC scores in both groups with a larger improvement in the treated patients. The MRI examination revealed no deterioration in the intervention group and 71% worsening (3 times total hip arthroplasty = THA needed) in the control group. These differences were statistically significant [33].

**Sen et al.** [34] and **Gangji et al.** [12] reported clinical trials, subdividing their ONFH patient populations into two groups (core decompression versus core decompression with application of BM mononuclear cells). **Sen et al.** reported of 40 patients with 51 affected hips (randomized, unblinded) and **Gangji et al.** reported of 13 patients with 18 affected hips (allocated, blinded). Both groups stated a larger improvement in clinical outcome scores in the intervention groups. Furthermore, the hip survival rate was higher in the cell treated cases. No complications and/or major side effects were described.

**Hernigou et al.** treated post-traumatic avascular necroses of the talus with either core decompression alone (control group, 34 cases) or with additional injection of BMAC (treatment group, 45 cases). The treatment group displayed a significantly lower percentage of collapse and need for arthrodesis surgery compared to the control group. Furthermore, the pain levels and the clinical symptoms showed a significant improvement in the BMAC group. The volume of repair in MRI was significantly larger in the intervention group as well [35].

**Yamasaki et al.** performed a clinical trial implanting a cell-seeded (BM mononuclear cells) interconnected porous calcium hydroxyapatite scaffold (IP-CHA) into the femoral head in 30 ONFH cases and retrospectively compared these with cell-free IP-CHA. The BM mononuclear cells were obtained from iliac crest BMAs, which were consecutively filtered and centrifuged with a yield of 40 ml BMAC containing approximately 1 × 10^9^ cells.

Clinical scores as well as mean pain scores improved in the treatment group (except for one case with need for THA), whereas the clinical scores decreased in the control group. The radiological imaging displayed no progression in 56.7%, mild collapse in 33.3% and a more extensive collapse progression in 10% of cases in the treatment group. In the control group, all cases showed progression (extensive progression in six of nine cases) with three cases requiring THA [36].

**Mao et al.** reported a case series with 62 patients with 78 cases of ONFH. The patients were treated with an intra-arterial injection of BMAC into the medial circumflex femoral artery. The group reported an overall failure rate of 7.7% with a mean conversion time to total hip replacement of 3 years. The HHS compared to baseline improved on each follow-up visit, but declined again after 36 months. An overall rate of radiological progression was noted in 43.59% of all patients [37].

A case series dealing with five cases (four patients) of osteonecrosis of the humeral head was published by **Makihara et al**. After obtaining autologous BMA from the patient’s ilium, centrifugation and processing, a BMAC graft was injected into the lesion site of the humeral head. Three of five cases showed a persistent stage of osteonecrosis (based on the Cruess classification) with no progression, whereas two cases deteriorated (one case required arthroplasty). The group reported reduction of pain and a variable development of range of motions after treatment [38].

In contrast to these mainly positive results in these studies, two trials reported contradictory findings.

**Pepke et al.** reported no difference in clinical or radiological outcome in a RCT, comparing core decompression alone versus core decompression plus BMAC, treating 24 patients (25 hips). The group stated, that BMAC application may not be beneficial in ONFH cases [39].

In the clinical trial of **Lim et al.**, 128 patients (190 ONFH hips) were examined retrospectively.

One group (107 patients, 159 ONFH) received multiple drilling and BMAC implantation whereas the other group (21 patients, 31 hips) was treated with core decompression and an additional bone graft. The group reported no difference in success rate (success defined as HHS with 75 points or higher and no need for further surgery) between both groups [40].

*Conclusion* most studies actually published deal with the use of MSCs in ONFH patients; other localizations are exemplary. The treatment options for ONFH are either nonoperative (e.g. nonweight bearing, bisphosphonate therapy, shock wave therapy and others) or operative. Among the operative options, core decompression is the best examined procedure, when searching the current literature [1]. The aim of all treatment considerations is to prevent the patients from total joint replacement. There is consent, that not all lesion sizes and localizations qualify for being treated with common therapeutic options. Therefore, there is a lot of clinical data testing and analyzing the application of MSCs or BMAC in cases of ONFH and comparing them with the actual standard of care treatment. Most of the results are positive, showing a beneficial effect. But, contrarily, some authors reported no improvement in the clinical and radiological outcome. To finally determine, whether the use of MSCs can improve the standard of care for ONFH treatment further prospective and randomized high-quality studies with larger population sizes, standardized control groups and protocols are needed, to reach the highest level of comparability between the different treatment options in ONFH. Additionally, a direct mode of action analysis of MSC treatment based on the known pathophysiology of osteonecrosis would contribute in further improvement of treatment strategies.

### Intervertebral disc degeneration/spinal cord injury/spinal fusion

The subdomains of spine surgery, as a specialized field in orthopedic surgery, mainly consist of decompression and fusion operations (either in an anterior, posterior or combined approach), disc replacements and minimally invasive procedures (such as sequestrectomies). Clinical trials for the use of human MSCs mainly concentrate on improving the neurological dysfunction after spinal cord injury, assisting the process of bony fusion after stabilization procedures or restoring a normal disc height and water content in degenerative disc disorders.

#### Intervertebral disc degeneration

The loss of integrity and physiological height of intervertebral discal structures lead to chronic back pain and may cause neurological symptoms in case of spinal cord or nerve root compression. Restricted quality of life is a consequence of this common disease. After a conservative approach consisting of adequate analgesia, manual therapy and physiotherapy, surgical procedures such as spinal fusion or intervertebral disc replacement therapies of affected segments are among current treatment concepts. In the following, clinical trials are discussed, analyzing the application of MSCs to regenerate physiological intervertebral disc structures.

#### Studies using MSCs

**Noriega et al.** performed a prospective study including 24 cases of degenerative disc disease (DDD) with an intact annulus fibrosus. They randomized their study population into two groups: one control group (sham injection in paravertebral muscles) and an intervention group (intra-discal injection of 25 × 10^6^ allogeneic BM-MSC). No AEs were described. In the intervention group, pain and disability scores significantly improved, whereas in the control group no significant changes were observed. The MRI showed no significant changes in both groups [41].

**Orozco et al.** published a case series including 10 patients with chronic lower back pain due to disc degeneration in the lumbar spine with an intact annulus fibrosus. After injection of autologous BM-MSCs (plastic adherence, 10 ± 5 × 10^6^ cells) in the nucleus pulposus area, a statistically significant improvement in pain and disability levels and in the physical component of the SF-36 score was described. In the MRI examination no significant changes in the disc height were observed, but the water content normalized (comparably to healthy discs). Key result of the findings of Orozco et al. was that the group found their procedure to be feasible and safe [42].

**Yoshikawa et al.** reported the treatment of two patients suffering from symptomatic intervertebral disc degeneration with an injection of cultivated autologous BM-MSCs seeded onto an autologous collagen sponge, implanted into the disc nucleus. In these patients the clinical symptoms as well as the pre-operatively discovered vacuum phenomena were reported to have improved after therapy. Additionally, the group observed a higher moisture content of the treated intervertebral disc in MRI [43].

*Conclusion* in conclusion, the use of MSCs in treating intervertebral disc degeneration, show a potential for regeneration. Further research is still in progress (e.g. a currently running randomized, placebo-controlled phase III trial investigating the intra-discal injection of allogenic mesenchymal precursor cells in patients with chronic lower back pain, ClinicalTrials.gov Identifier NCT01290367).

#### Spinal cord injury

The leading cause for spinal cord injuries are high-velocity traumata [44]. The possibility of MSCs to differentiate into cells of the neuronal lineage has been described and was the basis of several research efforts [6, 45].

#### Studies using MSCs

**Dai et al.** reported 40 patients with cervical spinal cord injury (complete and chronic), who were randomized into 2 groups: intervention group treated with injection of BM-MSCs (plastic adherence) via durotomy to injury site and control group treated without cell products.

In 50% of cases in the intervention group, an improvement in clinical parameters (motor function, sensory function and bladder function) was discovered, whereas in the control group no changes were observed. Additionally, 45% of the patients in the intervention group improved in AIS grading (American Spinal Injury Association impairment scale); no significant changes were seen in the control group.

Therefore, the group hypothesized that local MSC therapy was beneficial for neuronal recovery after spinal cord injuries [46].

In a RCT by **Cheng et al.** 34 cases of thoracolumbar spinal cord injury were subdivided into: (A) MSC transplantation, (B) rehabilitation therapy and (C) blank control (without any treatment). As a study agent, human umbilical cord-derived MSCs (hUC-MSCs) were used, which were harvested from full-term healthy new-borns, adherence selected and flow cytometry characterised. All patients in group A were treated with a computer tomography (CT) assisted MSC injection into the injured area of the spinal cord. In the cell treated group, all clinical parameters including motion, muscle tension and self-care ability showed statistically significant improvements. In contrast, the participants randomized into groups B and C did not show any significant changes in clinical outcome parameters. Furthermore, the urodynamic analysis (blank control group not included) displayed a significant increase of bladder capacity and a significant increase in detrusor pressure as well as an improvement in urinary flow and residual urine volume in the cell treated group. Group B showed a deterioration in all parameters. As conclusion, Cheng et al. declared the application of the hUC-MSCs to be an effective approach [47]. However, the study was not reported to be blinded.

**Hur et al.** examined 14 patients with spinal cord injury with different levels of injury severity: American Spinal Injury Association = ASIA A n = 12, ASIA B n = 1, ASIA D n = 1. In six cases the injury was located at cervical level, in one case at cervico-thoracic, in six cases at thoracic and in one case at lumbar level.

Autologous AD-MSCs (adherence selected) were administered intrathecally via lumbar spinal tapping. In follow-up controls, five patients showed motor function improvement. Two patients showed an improvement in anal sphincter function in follow-up examination. Furthermore, sensory improvement was observed in 10 patients (1 patient showed deterioration). Radiological and electrophysiological examinations showed no changes, apart from one case with somatosensory evoked potentials (SSEPs) improvements [48].

**Vaquero et al.** examined 10 patients with chronic spinal cord injury, who were treated with 4 subarachnoid injections of BM-MSCs. An improvement in sensory and motor function was reported. The activity of daily living scores significantly improved as well. Bladder and bowel function improved in the majority of patients (bladder dysfunction improvement in eight and bowel dysfunction improvement in seven patients). Furthermore, neurophysiological improvement throughout the follow-up period was reported for all patients [49].

#### Studies using BMAC

The prospective, randomized, single-blinded and controlled trial of **Chhabra et al.** investigated the effect of BMAC injections in 21 patients with acute traumatic spinal cord injury. Three groups were formed:

- *group A* BMAC injection in the surroundings of the injured spinal cord site (depth 5 mm from dorsal surface) via durotomy and sparing the arachnoid mater,
- *group B* BMAC injection intrathecally,
- *group C* control group (no application of BMAC).

All patients underwent surgical intervention through stabilization with or without decompression.

As key result, the authors concluded that the use of cell products was feasible but without any additional beneficial effect [50].

In a non-randomized clinical trial, **Yoon et al.** subdivided patients with complete spinal cord injury into 2 groups (control group, n = 13, treated with spinal decompression and anterior cervical fusion alone versus treatment group, n = 35, with additional injection of BMAC and granulocyte macrophage-colony stimulating factor = GM-CSF). They observed a development of neuropathic pain in 20% of patients in the intervention group and just 7.7% of patients developed similar symptoms in the control group, which was a non-significant result. The treatment group displayed neurological improvements in 29.5% (acute treatment subgroup), 33.3% (subacute) and in 0% (chronic group), respectively. The subdividing of “acute” (< 2 weeks), subacute (2 to 8 week) and chronic (> 8 weeks) refers to the time period between injury and cell treatment. Furthermore, they postulated, that the number of white blood cells in peripheral blood showed a significant effect on the neurological outcome (higher amount associated with larger improvement) [51].

**Park et al.** evaluated 6 patients with complete cervical spinal cord injuries (within 14 days). Five patients were treated with BMAC and GM-CSF and 15 with GM-CSF only. BMAC was applied via durotomy and injection around the contusion site. After surgery, GM-CSF was applied in 5 cycles (250 µg/m^2^ of body surface area). An improvement in neurological functions was described for all patients. No SAEs were reported [52].

*Conclusion* MSC applications in spinal cord injuries were declared superior to conventional treatment in some of the examined studies, whereas in others no improvements were found, which indicates a remaining need for clarification, if and which kind of cell therapy would benefit spinal cord injury patients, in particular with regard to neurological improvement. It has to be remarked, that in the case of neurological dysfunction due to traumatic injury, a development of neuronal tissue is expected whereas in contrast to the other here examined topics and subtopics the regeneration of tissue from the mesenchymal lineage shall be promoted by the applied MSCs or BMAC. The launch of the Stemirac Project in Japan, the intravenous injection of MSCs for patients suffering from spinal cord injury in the beginning of 2019, has been accompanied by a controversial discussion. The main point of critics as also published by D. Cyranoski is the lack of double-blinded efficacy studies [53]. Under the headline “slow down” [54] a further call for the necessity of performing a RCT was published in the same volume of the Nature Journal in January 2019 [54]. Answering the concerns, the Director-General of the Pharmaceutical Safety and Environmental Health Bureau of Japan’s Ministry of Health, Labour and Welfare stated ethical issues as main problem for further double-blinded studies [55]. We think, that further research and data analyses in terms of efficacy but also risk analyses are inevitable to evaluate this type of MSC use and to guarantee patients´ security.

#### Spinal fusion

Operative procedures to reach intersegmental spinal fusion are performed in cases of traumatic or osteoporotic fractures, degenerative spine disorders, deformities and others. The use of BMAC or MSCs aims at a faster and more effective bony fusion.

#### Studies using MSCs

**Fomekong et al.** performed a minimally invasive transforaminal lumbar interbody fusion (TLIF) applying autologous AD-MSCs (immunophenotyped, differentiation potential as marker for cell identity) in three patients (graft with DBM). They reported on no complications and observed an improvement in pain and disability scores. Two of three evaluated segments showed bony fusion in the CT scans [56].

#### Studies using BMAC

**Hart et al.** randomized 80 patients with degenerative diseases of the lumbar spine into two groups in a randomized, controlled, blinded study design: an intervention group treated with cancellous bone allograft chips and BMAC and a control group treated with cancellous bone allograft chips only. Both subpopulations received lumbar or lumbosacral postero-lateral fusion surgery. The intervention group showed a significantly better fusion rate compared to the control group as shown in plain radiography and CT scans. The authors concluded an overall improvement in bony healing after intersegmental spinal fusion when applying BMAC [57].

41 Patients with posterior spinal fusion due to DDD or instable thoracolumbar fractures were treated with autologous BMAC seeded onto beta-tricalcium phosphate granules (3–5 mm in diameter) by **Gan et al.** in a prospective non-controlled case series. A fusion rate of 95.1% resulted after 34.5 months in CT scans and three-dimensional reconstruction. Two patients with two-level spinal fusion showed one segmental non-union. No re-operation was necessary [58].

**R. Johnson** reported of 25 patients (24 finally evaluated) with either DDD, spondylolisthesis or lumbar spinal stenosis. They performed internal fixation and intersegmental spinal fusion augmented with either iliac crest bone grafts or BMAC mixed with cancellous allograft after randomization. Radiologically evaluated fusion rates (CT scans), showed no differences between both augmenting material [59].

**Odri et al.** published a case series including 15 patients with radicular and/or lumbar back pain. All patients were treated with postero-lateral lumbar arthrodeses. Intraoperatively, one fusion side was augmented with BMAC and the other side with non-concentrated BM, added to a graft of autologous bone mixed with granules of microporous biphasic calcium phosphate ceramics in both groups. No AEs were reported. CT scans were conducted 1 week and 3 months after surgery. Although the clinical parameters significantly improved and the authors did not experience any fusion failure, no significant difference in cortical bone volume was observed comparing both groups [60].

A retrospective analysis of 31 patients with posterolateral interbody fusion and transforaminal lumbar interbody fusion with a combined graft, consisting of BMAC and allograft bone chips, was conducted by **Ajiboye et al**. The graft was placed posterolaterally and into the interbody cages. The group reported of an overall bony fusion rate of 83.9% and an interbody fusion rate of 96.8%. One patient suffered from non-fusion with the need for further operative treatment. The clinical outcome parameters based on the modified Odom’s criteria displayed excellent or good results in 83.9% [61].

*Conclusion* concerning BMAC or MSC therapy in supporting bony fusion after stabilization procedures there is no clear evidence yet, regarding which approach would be most effective on bone healing. Regarding the here presented results, a clear comparison between the actual gold standard of autologous or allogeneic bone transfer and the additional or single use of MSCs at the fusion site is essential.

In all three fields further studies are needed to clearly verify the advantage of MSC or BMAC treatment compared to standard of care therapies.

### Muscle injuries

Apart from muscle tissue injuries resulting from high-velocity trauma, sports injuries and battlefield injuries, surgical procedures are another leading cause of skeletal muscle damage and loss. Systemic diseases such as Duchenne’s dystrophy, mitochondrial myopathy and glycogen storage diseases may also lead to a generalized skeletal muscle affection.

The surgical restoration of lost skeletal muscle volume can so far only be reached by muscle transfer surgeries, accompanied by a high donor site morbidity [62]. Injury-related loss of muscle function can only be partly and unsatisfactorily restored even by the most advanced surgical techniques. Therefore, the development of additional cell-based therapies for treating muscle injuries and defects is a substantial element of current research.

Our group investigated the safety and efficacy of placental-expanded adherent MSCs (PLX-cells; Pluristem Ltd., Haifa, Israel) on muscle regeneration in a prospective, randomized, double blinded and placebo-controlled phase I/IIa clinical trial. Twenty patients receiving THA were randomized into 3 groups: high dose (3.0 × 10^8^ PLX-cells), low dose (1.5 × 10^8^ PLX-cells) and placebo. Cells were locally injected into the injured gluteus medius muscle after the implantation of a THA. We found a significant increase of the contraction force of the abductor muscles in the low dose group after a follow up of 6 months, which was accompanied by an increase in muscle volume. This functional improvement could be correlated with a decrease of the early postoperative stress reaction as observed in immunological cellular and humeral biomarkers. The high dose group displayed an initial superior increase in muscle force but did not reach a significant higher force compared to the placebo group at final measurement, again demonstrating that the dosing is a critical issue in cell based therapies. No safety concerns were noted during the study and follow-up [63].

*Conclusion* based on the results of the presented clinical trial, recruitment in a phase III multicenter trial using PLX cells for improving mobility and mortality in femoral neck fracture arthroplasty patients is currently ongoing. The results of the presented study hold great hopes for establishing the use of MSCs in further therapeutic considerations dealing with muscle dysfunctions or injuries.

### Tendon/ligament/meniscus injuries and degeneration

Clinical data for the treatment of tendon, ligament or meniscus lesions and defects using MSCs are exceedingly rare in the current literature. Up to date, most of the studies and evaluations are in pre-clinical or animal model state [64–66]. Research focuses on many essential structures of the human musculoskeletal system, such as Achilles tendon, anterior cruciate ligament, medial collateral ligament and many more. To our knowledge, clinical trials using human MSCs have been published for rotator cuff tears, meniscus lesions, patellar tendinopathy and epicondylitis of the elbow so far.

#### Rotator cuff repair

#### Studies using MSCs

**Kim et al.** performed a clinical study with 70 cases (matched analysis) of symptomatic full-thickness rotator cuff tears. The subjects were subdivided into two groups: group A with arthroscopic rotator cuff repair and group B with arthroscopic rotator cuff repair with an additional injection of autologous AD-MSCs (plastic-adherence, characterization by differentiation potential, immunophenotyping). Arthroscopic repair was accomplished using a double-row suture bridge technique. MSCs were loaded onto a fibrin glue scaffold and applied to the tendon–bone junction, covering the repaired tendon. Pain and clinical outcome scores significantly improved in both groups with no significant difference between groups A and B. In evaluating the range of motion, only the external rotation and the forward flexion were significantly larger compared to baseline level; no significant differences between both groups were observed. However the structural outcome, measured via MRI, showed a statistically significant difference in the retear rate of 14.3% in injection and 28.5% in control group [67].

#### Studies using BMAC

A total of 90 patients with symptomatic rotator cuff ruptures were analyzed by **Hernigou et al.** in a matched cohort trial. 45 Patients were treated with an arthroscopic repair and application of BMAC at the tendon–bone junction and 45 patients with an arthroscopic repair alone as control group. After 10 years of follow-up, intact rotator cuffs could be diagnosed in 87% of cases in the cell treated group and in 44% of patients enrolled in the control group. It is of note that within the cell treated group only one tendon of the rotator cuff was affected when a retear had occurred, whereas within the control group up to two or three tendons were affected in retear patients. The group also found a correlation between the count of MSCs injected and the healing rate. The higher the number of implanted cells, the lower the failure rate of arthroscopic repair and the faster the healing. The authors concluded, that there were clear benefits of injecting BMAC during surgical rotator cuff repair [68].

**Ellera Gomes et al.** reported of a case series of 14 patients suffering from rotator cuff lesions. All of the studied subjects received rotator cuff repair with augmentation via Ficoll sorted cells through injections in ruptured tendon and footprint. MRI analysis after 12 months showed tendon integrity in 100% of patients. The group reported improved clinical outcome scores and healed tendon in all but one patient at the end of second year after surgery. The patient had to undergo revision surgery due to an increase in pain and a loss of strength. The authors concluded the application of BMAC to be beneficial in rotator cuff repairs compared to current literature and standard of care [69].

#### Others

#### Studies using MSCs

A randomized, double-blinded and controlled study with 55 subjects after partial medial meniscectomy was published by **Vangsness et al.** The patients were intraarticularly injected with:

- *group A* 50 × 10^6^ allogenic BM-MSCs,
- *group B* 150 × 10^6^ allogenic BM-MSCs,
- *group C* vehicle control group (treated with hyaluronic acid alone).

The cells were harvested from adult human unrelated donors (Osiris Therapeutics, Columbia, Maryland). Nine SAEs were reported but declared to be unlikely associated with the application of MSCs by blinded investigators. Pain scores improved with statistical significance between MSC groups and control group. A development of subchondral sclerosis and formation of osteophytes, indicating further development of arthritic changes, was described in 21% of patients in the control group compared to 6% of patients treated with intra-articular MSC application. The procedure was judged by the authors to be safe and beneficial but without further improvements when using higher doses of MSCs [70].

**Lee et al.** treated 12 cases of chronic lateral epicondylosis with allogenic AD-MSCs (isolated from healthy donors, characterized by karyotype, cell surface markers and morphology) in two different dosages (high-dose 10^7^ cells and low-dose 10^6^ cells). MSCs were injected on a fibrin glue scaffold into the largest hypoechoic lesion under guidance of ultrasound imaging. No SAEs were reported. Pain score and clinical outcome assessments improved without significant differences between both groups, except for the high-dose group showing a faster pain release than the low-dose group. The objective outcome measurement through ultrasound revealed a decrease in lesion size without any significant difference comparing both groups. The authors declared their procedure to be safe and efficient, yet the study didn’t follow a controlled protocol [11].

#### Studies using BMAC

**Pascual-Garrido et al.** presented their findings of eight patients with chronic patellar tendinopathy in a small, non-controlled study. The group treated the study subjects with an ultrasound-guided injection of BMAC into the lesion site. No complications were reported and the clinical outcome scores improved. Grading based on the ultrasound diagnostics improved as well in all but one patient [71].

*Conclusion* current concepts for treating tendon and ligament pathologies are widespread and depend on the cause of rupture, lesion size and patient’s individual health status. Especially for chronic ruptures of the rotator cuff, a high failure rate of 30–94% in chronic lesions even with recently developed surgical techniques is described in the current literature [72]. The use of MSCs in orthopedic diseases described above shows potential to improve healing rates and clinical recovery of patients. However, clinical evidence is still weak and further prospective, randomized and blinded studies with larger patient numbers and suitable control groups will be necessary to clarify the real benefit of MSC applications in tendon-, ligament- and meniscus pathologies. Additionally, a clear analysis of the different dose–response correlations is essential due to the yet inconsistent application.

## Osteochondral defects

Osteochondral defects in joints occur mostly after trauma or due to osteonecrosis. Lesions in the cartilage can lead to pain and restricted mobility. Therefore, a lot of surgical interventions like microfracturing, autologous chondrocyte implantation (ACI), osteochondral autograft transplantation and others) were developed to treat these patients [73]. MSC therapy has been pioneered in this field by the treatment of osteochondral defects with autologously harvested and expanded chondrocytes. Studies investigating the use of MSCs remain scarce. Most studies mainly concentrate on cartilage lesions of the knee joint with few exceptions addressing other joints.

### Uncontrolled studies

**Giannini et al.** examined 48 patients with focal osteochondral lesions of the talar dome.

Autologous BM-MSCs (Ficoll selected and tested for differentiation capability) on two different scaffolds, one of porcine collagen powder, the other of hyaluronic acid membrane (both augmented with platelet-rich fibrin gel), were inserted into the lesion via ankle arthroscopy. Clinical outcome scores improved without any relevant difference between both scaffolds. MRI and arthroscopic inspection showed defect filling and a regenerated cartilage layer with two cases of hypertrophic tissue formation [74].

A case series with 10 patients suffering from isolated articular cartilage defects in the knee joint, treated with allogenic BM-MSCs (density gradient and adherence isolated, immunophenotyped) mixed with autologous chondrons in fibrin glue was published by **de Windt et al.** in 2016. The authors observed a significant improvement in clinical outcome scores and pain and a complete filling of former defects in MRI. Nine patients received an arthroscopic reevaluation, showing six cases of normal tissue repair and three cases of nearly normal tissue repair macroscopically [75].

**Haleem et al.** reported five patients with a full-thickness cartilage defect of the femoral condyle treated with microfracturing, followed by autologous BM-MSC (Ficoll and adherence selected, characterized by flow cytometry and gene expression) transplantation (on a platelet enriched fibrin glue gel) into the defect site covered by an autologous periosteal flap. The authors described a statistical significant improvement in clinical symptoms. MRI examination revealed three cases of complete defect fill and congruity of articular surface and two cases with partially filled defects [76].

**Wakitani et al.** published two case series of osteochondral lesions treated with MSCs:

Three patients (five knees) were treated with autologous BM-MSCs (adherence selected, surface marker characterized) seeded onto a collagen sheet graft with an autologous periosteal flap for articular cartilage defects of the patellofemoral joint. The group described a coverage of the former defect in either arthroscopy or MRI and an improvement in clinical symptoms after 17–27 months of follow-up [77]. In another case series, two patients with patellofemoral cartilage defects were included. Autologous BM-MSCs seeded onto a collagen gel graft were transferred into the lesion after microperforation of the subchondral bone and coverage was achieved by an autologous periosteum flap. Clinical symptoms improved, arthroscopic reevaluation revealed the coverage of the defects and the histological analysis showed fibrocartilaginous tissue development [78].

### Controlled studies

Five studies compared the use of MSCs or BMAC with microfracturing or ACI.

**Koh et al.** published a follow-up study of their prospective randomized trial of 80 patients with symptomatic single cartilage lesions of the femoral condyle. One group of patients received treatment with microfracturing alone, whereas the other group was treated with microfracturing and implantation of autologous AD-MSCs (adherence selected, immunophenotyped) in a fibrin glue graft. MRI revealed a complete cartilage coverage of the defect in 45% of the microfracturing group, compared to 65% in the microfracturing plus MSC group. Pain scores improved in both groups but with a significantly better result in the microfracturing plus MSC group compared to the controls. Arthroscopic findings showed no relevant intergroup differences [79].

A comparison of 70 symptomatic full-thickness chondral lesions of the knee joint treated with either arthroscopic microfracturing and intra-articular injection of autologous BM-MSCs (selected via density gradient and immunophenotyping) or application of BM-MSCs in an open approach beneath a periostal flap was conducted by **Lee et al.** Improved clinical outcome scores were observed in both groups. Pain scale improved without any relevant intergroup difference. The BM-MSC injection group showed significant better results in two clinical outcome scores and MRI showed a good fill and tissue integration in the BM-MSC injection group. Findings of the open approach were not displayed [80].

**Nejadnik et al.** reported 72 patients with full-thickness cartilage lesions in the knee joint. 36 of them received an ACI procedure, whereas autologous BM-MSCs (adherence selected) in cell sheets were transplanted in the other 36 cases. The group described the clinical outcome scores to have significantly improved in all groups [81].

The following studies are using BMAC:

**Gobbi et al.** subdivided 37 patients with full-thickness chondral lesions of the patellofemoral joint into two groups: group A received matrix-induced ACI (MACI) whereas group B received an application of BMAC in hyaluronic acid. Pain score and clinical outcome assessments significantly improved in both groups without relevant differences except in one sub-score (International Knee Documentation Committee subjective score showed significant improvement). MRI showed complete or near complete filling of the defects in 81% of the patients in the BMAC group and in 76% in the ACI group. Arthroscopic biopsy results were comparable between both groups as well [82].

**Giannini et al.** compared 81 patients with focal osteochondral lesions of the talar dome receiving either ACI in an open approach (with periostal flap), arthroscopic ACI (hyaluronic acid scaffold) or BMAC administration on collagen powder or hyaluronic acid membranes. The authors described a statistically significant improvement in clinical outcome scores in all subgroups with no relevant differences between them. MRI showed a nearly complete integration of regenerated tissue in 76% of all subjects [83].

*Conclusion* according to the current literature, the actual concepts in treatment of osteochondral lesions include osteochondral grafting, procedures to stimulate the subchondral BM, osteochondral scaffolds and cell therapies. Some of the strategies reveal relevant disadvantages such as donor site morbidity and surgical exposure [84]. The use of human MSCs in osteochondral defects has yielded promising results in clinical and objective outcome parameters in several trials. Clinical research within this field has progressed more than in other fields. Nevertheless, further and larger studies will be necessary to proof the benefit and advantages of MSCs compared to other well-accepted cell-based and cell-free treatment options in osteochondral defect treatment. In summary, an advantage when compared to the gold standard of chondrocyte transplantation has not yet been clearly demonstrated.

## Osteoarthritis

OA limits patient’s quality of life due to restriction of mobility, increasing pain and decreasing comfort. OA is defined by a loss of articular cartilage and a degenerative remodeling of the subchondral bone and periarticular soft tissues. Causes for OA range from malalignment, crystalline diseases, history of trauma, systemic inflammatory diseases and multiple other diseases affecting the joint, yet idiopathic arthritis is still the leading entity [85].

The use of MSCs in the treatment of OA mainly concentrates on the knee joint in current clinical trials.

### Studies using AD-MSCs or SVF

When reporting AD-MSC related studies, the type of the investigational product needs to be clearly defined. In common literature the most important distinction is that of SVF (stromal vascular fraction) and specifically AD-MSCs. The SVF, mostly harvested through lipoaspiration of subcutaneous fat tissue, contains blood vessels and collagen fibers next to the stromal cell fraction. In further processing, cultivation and selection methods, isolated AD-MSCs can be selected [86].

The groups of **Koh et al.**, **Jo et al.** and **Kim et al.** and **Pers et al.** investigated the use of AD-MSCs.

**Jo et al.**, subdivided nine patients with knee OA into three treatment groups: all patients received arthroscopic surgery and injection of autologous AD-MSCs (analyzed for cell number, viability, purity and phenotype) in either low-dose (1.0 × 10^7^ cells), mid-dose (5.0 × 10^7^ cells) or high-dose (1.0 × 10^8^ cells). In a second phase of the study, nine additional patients were treated with high-dose AD-MSCs. No control group was analyzed. Western Ontario and McMaster Universities osteoarthritis index (WOMAC) clinical outcome score and pain significantly improved in the high-dose group compared to the other two groups, whereas the Knee Society clinical rating system (KSS) score increased in low-dose and high-dose groups. MRI revealed a decrease in cartilage defects and an increase in cartilage volume in the high-dose group but no significant changes in the other groups [87]. In a follow-up study, the group described the safety and efficacy of the procedure but stated concerns about the persistence of positive effects [88].

The group of **Pers et al.** published their bicentric, prospective and uncontrolled phase I study including 18 patients with knee OA (grades three to four on Kellgren and Lawrence scale). The patients were randomized in three different dosage arms: intra-articular injection of 2 × 10^6^, 10 × 10^6^ or 50 × 10^6^ cells. The autologous AD-MSCs were harvested via liposuction and adherence testing as well as phenotyping. In a 6 months follow up period, the procedure was declared to be safe. An improvement in clinical outcome parameters was shown in all arms but with statistical significance only for the low dose treatment arm [89].

A statistically significant improvement in all clinical outcome assessments and pain without any difference between 25 patients with knee OA treated with AD-MSC injections, harvested from the infrapatellar fat pad (enzymatically isolated) and 25 matched patients, receiving intra-articular PRP injections, was described by **Koh et al.** [90].

**Koh et al.** examined 44 patients with isolated medial knee compartment OA. A high-tibial osteotomy was performed and additionally one group received PRP intra-articular injections and the other group received PRP and autologous SVFs (characterized by immunophenotype and differentiation potential) injections intra-articularly. Clinical outcome scores and pain improved, showing a significantly more pronounced pain decrease in the PRP plus SVF group. A second-look arthroscopy revealed better healing rates of the articular cartilage layer in the cohort treated with PRP plus SVF injections [91].

Another study from the **same group** with 30 patients suffering from knee OA, treated with arthroscopic injections of autologous SVFs plus PRP resulted in significant improvements in pain and clinical outcomes [92].

Another study using SVFs from the patient’s infrapatellar fat pad with a retrospective analysis of 18 patients suffering from knee OA, revealed significant improvements in clinical outcome and pain scores as well. MRI showed an improvement in cartilage tissue based on the whole-organ MRI score, WORMS [93].

The **same group** retrospectively evaluated 37 patients with knee OA treated with autologous SVFs derived from the infrapatellar fat pad derive and observed all clinical outcome scores to be significantly improved depending on patient’s weight and lesion size [94].

Two retrospective analyses were published by **Kim et al.** The group examined 55 [95] and 56 knees [96] with OA, using either fibrin glue as scaffold for the SVF injection or SVF alone. Improvements in the clinical outcome were reported. Furthermore, outcomes were correlated to patient’s weight, age and defect size [95, 96].

### Studies using BM-MSCs

The group of **Vega et al.** analyzed allogenic BM-MSC OA treatment in a blinded RCT with 30 patients. When compared with the control group receiving hyaluronic acid, the MSC-treated patients had a larger improvement in clinical outcome and pain. The authors reported a significantly improved MRI morphology (Poor cartilage index) as well [97].

**Lamo-Espinosa et al.** randomized 30 patients with knee OA into 3 treatment groups: Intra-articular injection of hyaluronic acid, low-dose BM-MSC injections and high-dose BM-MSC injections (autologous, Ficoll and adherence selected, flow cytometry characterized). Blinding was not reported. No significant changes were seen in the control group. In both MSC groups, pain levels significantly decreased and the WOMAC clinical outcome assessment significantly improved in the high-dose group. The overall range of motion of treated joints also improved in both MSC groups with a faster recovery rate in the high-dose group [98].

**Gupta et al.** presented a placebo (plasma-lyte A) controlled study, analyzing the effect of the Stempeucel® product (pooled, ex vivo expanded allogenic BM-MSCs, Stempeutics Research Bangalore, India) in patients suffering from knee OA in four dose levels after intra-articular injection. Pain levels decreased and clinical outcome improvements were more pronounced in the low-dose MSC group. MR and X-ray imaging did not reveal relevant changes [99].

**Orozco et al.** treated 12 knee OA patients with autologous BM-MSCs (Ficoll and adherence selected) through intra-articular injections. Clinical outcome scores and pain levels improved, whereas the SF-36 questionnaire did not. The MRI-based Poor cartilage index improved significantly after treatment [100]. Furthermore, the group conducted a 2 year follow-up of the study cohort, revealing maintained pain reduction and improved clinical outcomes. The cartilage index in MRI improved further within the follow-up period [101].

A nonrandomized, dose-evaluating phase I/II trial with 12 patients suffering from knee OA was published by **Chahal et al.** The patients were subdivided into four group, all of them receiving intraarticular injections of autologous BM-MSCs (Ficoll, density, immunophenotypisation, differentiation potential, gene expression) but in different dosages.

- group 1 receiving 1 × 10^6^ BM-MSCs,
- group 2 receiving 10 × 10^6^ BM-MSCs,
- group 3 receiving 50 × 10^6^ BM-MSCs,
- group 4 with mixed dosages (one patient received 1 × 10^6^ BM-MSCs, one patient received 10 × 10^6^ BM-MSCs and the last patient within the group received 50 × 10^6^ BM-MSCs).

As primary endpoint, no SAEs were detected throughout the study process. As secondary endpoints, the Knee Injury and Osteoarthritis (KOOS) as well as the WOMAC score were used. The clinical outcome parameters (pain scale and functional outcome, except KOOS sports subscale) in the 50 × 10^6^ BM-MSCs group showed the most relevant improvements (in number of patients), compared to the other dosage groups. The MRI scan analyses at 6 and 12 months after treatment showed no significant changes in all group. Additionally, the study group performed a biomarker analysis. Here, the cartilage catabolic factors increased in blood testing in patients received injection of 1 × 10^6^ BM-MSCs, whereas the other dosage groups revealed no significant changes. For further biomarker analysis we refer the reader to the original article [102].

The group of **Emadedin et al.** analyzed six patients with knee OA. After intra-articular treatment with autologous BM-MSCs (adherence selected, characterized via surface markers), pain reduction, increase in range of motion and walking distance as well as an increase in cartilage thickness were described [103]. The **same group** performed a long-term follow-up study with three groups (six patients each): knee, ankle and hip OA. In all cases, BM-MSCs were injected intra-articularly. An improvement of walking distance, clinical outcome scores and pain levels were observed. Radiological improvement based on MRI was observed in all patients. The group reported no SAEs and no tumor or neoplastic changes in the follow-up period [104].

Further case series, using BM-MSCs (intra-articular injection) for patients with knee OA were presented by **Centeno et al.** [105], **Soler et al.** [106] and **Davatchi et al.** [107, 108]. All of them reported improved pain and clinical outcome scores. Centeno et al. and Soler et al. furthermore examined a regenerative trend in MRI morphology in their studies.

Two studies examined the benefits of MSC treatment in addition to a high tibial osteotomy:

**Wakitani et al.** conducted a clinical trial with 24 patients suffering from medial unicompartmental OA of the knee. Two groups were compared: group A treated with cell-seeded (autologous BM-MSCs, adherence selected) collagen gel and group B with spongialization for cancellous bone exposure, periostal flap and a collagen sheet. For the MSC-treated group, significantly better results in the mean grading score (including arthroscopic and histological elements and subscores) were described [109].

Significantly improved clinical outcome scores and MRI scores (complete cartilage coverage in 32% of patients treated with BM-MSCs (characterized by morphology and flow cytometry) compared to 0% in the control group) were published by **Wong et al.** They compared two prospective randomized groups, including a total of 56 patients with medial unicompartmental OA. Patients were either treated with intra-articular autologous BM-MSC injections or with intra-articular hyaluronic acid injections. Patients were randomly assigned with staff members blinded and patients unblinded for treatment [110].

### Studies using MSCs of other origins

The group of **Matas et al.** analyzed the results of 26 patients suffering from knee OA in their phase I/II trial. The patients were randomized into three different groups:

- control group (receiving HA intraarticular injection two times),
- MSC-1 group (receiving 20 × 10^6^ UC-MSCs as intraarticular injection for one time and one-time placebo injection),
- MSC-2 group (receiving two times of intraarticular injection of 20 × 10^6^ UC-MSCs).

The cells were adherence tested. As primary endpoint of the clinical investigation, the procedure was declared to be safe. Furthermore, the pain levels were significantly reduced comparing MSC-2 group and control group. The MSC-1 group showed improvements up to the nine months endpoint and then reaching similar symptoms comparing with the control group. The MSC-2 group showed a continuing improvement of symptoms up to the final endpoint. There were no structural changes in MRI scans detected in all groups [111].

*Conclusion* multiple clinical research activities have been undertaken in the past years dealing with the use of MSCs in osteoarthritic patients. The overall aim of all studies is to prevent or delay total joint replacement. In summary, the results from the listed and described studies indicate that the application of human MSCs in OA could be an add-on for established therapeutic options in the future. However, also in this quite well developed research area, there is a lack of large, randomized, blinded studies.

## Summary and conclusion

There are only few studies published so far dealing with the clinical translation of the huge amount of pre-clinical data and results concerning MSC applications in musculoskeletal disorders.

The implementation of MSC therapy as standard treatment is so far complicated by a lack of standardization of the MSC handling and application itself, by a lack of proper prospective randomized trials with well-defined patient stratification and characterization and also, to some extent, by regulatory hurdles.

On the *product side* this means that there is no consensus on the details on cell harvest, cell selection and cell cultivation. Furthermore, there is a lack of in depth characterization of the therapeutic cells despite an abundance of available laboratory methods allowing a better understanding of the different cell products on a molecular level. Therefore, we encounter the problem that a comparison of different trials—even if these are RCTs—is hard to achieve.

On the *patient side*—apart from the lack of large RCTs—we are confronted with a lack of thoroughly characterized cohorts and above all with a lack of biomarker studies accompanying the trials aimed at revealing the mode of action within patients. These will be critical in the understanding of cell-based therapies since the main mechanisms have been revealed to be systemic and endo-/paracrine, independent of the respective indication [112].

A major role in all MSC-based trials seems to be the interaction of the investigational products and the immune system, which has been recognized as a regeneration system being involved in all processes where tissue homeostasis is compromised and healing takes place [113]. Hence, biomarker studies also have to address the impact of cell therapies on the immune system in order to reveal not only safety aspects but also a deeper understanding of systemic mechanisms of actions of the applied cells [114].

In stroke patients for example, it has been shown that MSC infusion does only effect the penumbra positively if the cells are applied within a certain time window [115]. This can also be expected in musculoskeletal indications, since all traumatic, degenerative and also complex diseases pass defined phases of inflammation, which exhibit different characteristics and different susceptibilities to cell therapeutic approaches. The only study found, investigating the ideal time frame for cell therapy in orthopedic disorders was published by Yoon et al. [51]. The group reported of higher rates of neurological improvement after cell therapy in the acute and subacute period after spinal cord injury, whereas in chronic disease states no benefit from cell application was noticed [51].

One important point that has to be raised is that specifically in musculoskeletal indications a lot of MSC products are combinatory products using cells in combination with a scaffold temporarily or permanently replacing bone, tendons or other structures. In bone, e. g., scaffolds can provide early stability and also by themselves improve mostly osteoconductive healing whereas the cells are used as the active part to induce bone healing. Apart from the choice of the ideal scaffold, whose biological but also mechanical properties will determine its function, a thorough preclinical characterization of the interplay between cells and the scaffold is an absolute prerequisite before introducing the products into the patients [25, 116, 117]. In combination with the substantial regulatory requirements this can be a challenge for academic institutions to provide, which explains the fact that not many products have reached the market yet, despite a relevant clinical need for their implementation.

Safety issues concerning MSC based therapies, which have been the focus of interest in all earlier trials have been mitigated by a lot of data showing that no relevant product related side effects have evolved during the follow up of the performed trials in all indications. In a multi-center analysis of 2372 patients treated with MSC-based percutaneous injections in orthopedic disorders, Centeno et al. reported 325 AEs (in 12.1% of all patients) with 36 severe AEs (SAEs, 1.5% of patients) with a follow up time of one month up to 8.8 years (2.2 years mean). The most common AEs were pain post-procedure or due to the degenerative disease itself. SAEs mainly consisted of vascular events, neurologic events and neoplasms. Especially, the incidence of neoplastic events following application of MSCs was shown not to be higher in treatment groups compared to the general population. Overall, multi-center trials showed that “pain” possibly linked to the application of MSCs is the most common AE [118, 119].

In conclusion, a lot of work still has to be done before finally adding MSC therapy to standardized clinical protocols. However, we have already reached a point where certain therapies can be identified as promising candidates for a translation into clinical routine either as a stand-alone treatment or an additive therapy to standard procedures.

As perspective, clinicaltrials.gov currently displays 116 running clinical trials, when searched for the terms “mesenchymal stromal cells” (access 10/2018) of which 19 studies deal with orthopedic diseases.

## Appendix

See Table 1.

**Table 1:** Overview of included studies concerning indication, number of patients enrolled (regardless different locations or if both sides were treated, control group in total included), cell product, different scaffolds or relevant supplements and author(s)

Main indication	Special indication	Patients	Cell product	Dosage	Carrier/scaffold	Author(s)
Bone defects/fracture	Bony defects in loosening of total hip arthroplasty	18	BM-MSC	15 ± 4.5 × 10^6^	Ultraporous beta-tricalcium phosphate absorbale sponge (Vitoss, Stryker®)	Šponer et al. [17]
	Tibial fractures	24	BM-MSC	Min. 5 × 10^6^	Composite graft (DBM, PRP)	Liebergall et al. [18]
	Non-unions in upper extremity	8	BM-MSC	1–4 × 10^6^ cells/2 ml	Blood clots (autologous plasma gel and CaCl_2)_	Giannotti et al. [19, 20]
	Musculoskeletal neoplasia/pseudoarthrosis	6	AD-MSC	Mean of 16 ± 4 × 10^6^	Three dimensional graft (DBM)	Dufrane et al. [21]
	Large bone defects	4	BM-MSC	2.0 × 10^7^ cells/ml	Microporous cylinders (hydroxyapatite vs ceramic)	Marcacci et al. [22]
	Substantial bone loss	3	Osteo-progenitor cells	Not given	Microporous hydroxyapatite scaffold	Quarto et al. [23]
	Tibial shaft non-union	1	BM-MSC	5 × 10^6^	Calcium sulphate pellets	Bajada et al. [24]
Osteonecrosis	ONFH	100	BM-MSC	2 × 10^6^	–	Zhao et al. [28]
	ONFH	24	BM-MSC	10^6^ cells/ml × 2 ml	Tantalum rod	Zhao et al. [29]
	ONFH	10	BM-MSC	0.5–1.0 × 10^8^	Beta tricalcium phosphate granules and vascularized bone graft	Aoyama et al. [30]
	ONFH	9	UC-MSC	5 × 10^6^–1 × 10^7^/ml (10 ml total)	–	Chen et al. [31]
	ONFH	40	Mono-nuclear cells/BMA	1.1 × 10^8^	–	Rastogi et al. [32]
	ONFH	18	BMAC	5 ± 2 × 10^8^ Mononuclear cells	–	Tabatabaee et al. [33]
	ONFH	40	Mono-nuclear cells	5 × 10^8^	–	Sen et al. [34]
	ONFH	13	Mono-nuclear cells	2.0 ± 0.3 × 10^9^ Leukocytes; 92 ± 9/10^7^ cells (fibroblast colony-forming units)	–	Gangji et al. [12]
	Post-traumatic avascular talus necrosis	79	BMAC	124 × 10^3^ Cells	–	Hernigou et al. [35]
	ONFH	30	Mono-nuclear cells	1 × 10^9^ Cells	Interconnected porous calcium hydroxyapatite scaffold	Yamasaki et al. [36]
	ONFH	62	BMAC	1.26 ± 0.5 × 10^9^ Cells	–	Mao et al. [37]
	Osteonecrosis of humeral head	4	BMAC	1125 Cells	–	Makihara et al. [38]
	ONFH	24	BMAC	18.9 ± 3.1 × 10^6^/ml (after concentration: 6.3-fold)	–	Pepke et al. [39]
	ONFH	128	BMAC	1.69 × 10^7^ CD34+ cells	Bone graft (control group only)	Lim et al. [40]
Spinal disorders	Intervertebral disc degeneration	24	BM-MSC	25 × 10^6^	–	Noriega et al. [41]
	Intervertebral disc degeneration	10	BM-MSC	10 ± 5 × 10^6^	–	Orozco et al. [42]
	Intervertebral disc degeneration	2	BM-MSC	10^5^ Cells/ml	Autologous collagen sponge	Yoshikawa et al. [43]
	Cervical spinal cord injury	40	BM-MSC	8 × 10^5^/µl (25 µl total)	–	Dai et al. [46]
	Thoracolumbar spinal cord injury	34	UC-MSC	4 × 10^7^	–	Cheng et al. [47]
	Spinal cord injury (diff. locations)	14	AD-MSC	9 × 10^7^	–	Hur et al. [48]
	Chronic spinal cord injury	10	BM-MSC	120 × 10^6^ in total	Autologous plasma	Vaquero et al. [49]
	Acute traumatic spinal cord injury	21	BMAC	2 × 10^8^ per 1.8 ml	–	Chhabra et al. [50]
	Spinal cord injury	48	BMAC	2 × 10^8^	GM-CSF add	Yoon et al. [51]
	Cervical spinal cord injury	6	BMAC	1.1 × 10^6^/µl (total 1.8 ml)	GM-CSF add	Park et al. [52]
	Minimally invasive transforaminal lumbar interbody fusion	3	AD-MSC	20 ± 4 × 10^6^ per three flasks	DBM graft	Fomekong et al. [56]
	Degenerative diseases of lumbar spine (operation)	80	BMAC	1–10 × 10^6^/l nucleated cells	Cancellous bone allograft chips	Hart et al. [57]
	Degenerative disc disease (posterior spinal fusion operation)	41	BMAC	44.5 ± 15 × 10^6^/ml nucleated cells	Beta-tricalcium phosphate granules	Gan et al. [58]
	Degenerative disc disease/spondylolisthesis/lumbar spinal stenosis (operation)	25 (24)	BMAC	17.7 ± 12.3 × 10^6^ Mononuclear cells/ml	Cancellous allograft	Johnson [59]
	Posterolateral lumbar arthrodeses	15	BMAC	22.4 ± 18 × 10^6^/ml nucleated cell	Autologous bone + granules of microporous biphasic calcium phosphate ceramics	^Odri et al.^[60]
	Posterolateral interbody fusion/transforaminal lumbar interbody fusion	31	BMAC	Not given exactly	Allograft bone chips	Ajiboye et al. [61]
Muscle injury	Muscle trauma due to operative procedure	20	Placental-expanded MSC (PLX-PAD)	1.5 × 10^8^ vs. 3 × 10^8^	–	Winkler et al. [63]
Soft tissue injuries/degeneration	Rotator cuff tears	70	AD-MSC	4.46 × 10^6^	Fibrin glue	Kim et al. [67]
	Rotator cuff ruptures	90	BMAC	51,000 ± 25,000	–	Hernigou et al. [68]
	Rotator cuff lesions	14	BMAC	3.81 × 10^8^ Mononuclear cells, 5.65 × 10^6^ CD34+ cells	–	Ellera Gomes et al. [69]
	Partial medial meniscectomy	55	BM-MSC	50 × 10^6^ vs. 150 × 10^6^	Hyalurone, albumin, PlasmaLyte A	Vangsness et al. [70]
	Chronic lateral epicondylosis (elbow)	12	AD-MSC	10^6^ versus 10^7^/ml	Fibrin glue	Lee et al. [11]
	Chronic patellar tendinopathy	8	BMAC	30 × 10^3^ (Total cell amount)	–	Pascual-Garrido et al. [71]
Osteochondral defects	Talar osteochondral lesion	48	BM-MSC	Not given	Porcine collagen powder versus hyaluronic acid membrane (both + platelet-rich fibrin gel)	Giannini et al. [74]
	Cartilage defects in knee joint	10	BM-MSC	1.5–2 × 10^6^ Cells/ml	Autologous chondrons in fibrin glue	de Windt et al. [75]
	Cartilage defects of femoral condyle	5	BM-MSC	Not given (inoculum density: 2 × 10^6^/cm^2^)	Platelet enriched fibrin glue gel	Haleem et al. [76]
	Osteochondral lesions of knee joint	3	BM-MSC	5 × 10^6^/ml	Collagen sheet	Wakitani et al. [77]
	Patellofemoral cartilage defects	2	BM-MSC	1.8 × 10^7^ and 1.4 × 10^7^	Collagen gel	Wakitani et al. [78]
	Cartilage lesions of femoral condyle	80	AD-MSC	4.97 × 10^6^	Fibrin glue	Koh et al. [79]
	Chondral lesions of knee joint	70 knees (pat.-nr. unclear)	BM-MSC	10 × 10^6^	Hyalurone add	Lee et al. [80]
	Cartilage lesions in knee joint	72	BM-MSC	Ca. 2 × 10^6^ cells/cm^2^ (four cell sheets)	Cell sheets	Nejadnik et al. [81]
	Chondral lesions of patellofemoral joint	37	BMAC	Not given	Hyalurone	Gobbi et al. [82]
	Osteochondral lesions of talar dome	81	BMAC	Not given	Collagen powder versus hyalurone membrane	Giannini et al. [83]
Osteoarthritis	Knee OA	9	AD-MSC	1 × 10^7^ vs. 5 × 10^7^ vs. 1 × 10^8^	–	Jo et al. [87, 88]
	Knee OA	18	AD-MSC	2 × 10^6^ vs. 10 × 10^6^ vs. 50 × 10^6^	–	Pers et al. [89]
	Knee OA	25	AD-MSC	1.89 ± 10^6^	PRP add	Koh et al. [90]
	Medial knee OA	44	SVF	4.83 × 10^7^ SVF cells	PRP add	Koh et al. [91]
	Knee OA	30	SVF	4.16 × 10^7^ SVF cells	PRP add	Koh et al. [92]
	Knee OA	18	SVF	1.18 × 10^6^ “stem cells”	PRP add	Koh et al. [93]
	Knee OA	37	SVF	3.8 × 10^6^ “stem cells”	–	Koh et al. [94]
	Knee OA	49	SVF	4.6 × 10^7^ SVF cells	Fibrin glue product	Kim et al. [95]
	Knee OA	54	SVF	4.2 × 10^7^ SVF cells	Fibrin glue product	Kim et al. [96]
	Knee OA	30	BM-MSC	40 × 10^6^	–	Vega et al. [97]
	Knee OA	30	BM-MSC	10 × 10^6^ vs. 100 × 10^6^	Hyalurone	Lamo-Espinosa et al. [98]
	Knee OA	60	Stempeucel® (pooled, ex vivo expanded allogenic BM-MSC)	25 × 10^6^ vs. 50 × 10^6^ vs. 75 × 10^6^ vs. 150 × 10^6^	–	Gupta et al. [99]
	Knee OA	12	BM-MSC	40 ± 1 × 10^6^	–	Orozco et al. [100, 101]
	Knee OA	12	BM-MSC	1 × 10^6^ vs. 10 × 10^6^ vs. 50 × 10^6^	–	Chahal et al. [102]
	Knee OA	6	BM-MSC	20–24 × 10^6^	–	Emadedin et al. [103]
	Knee OA/ankle OA/hip OA	18	BM-MSC	5 × 10^5^ cells/kg/bw	–	Emadedin et al. [104]
	Knee OA	1	BM-MSC	22.4 × 10^6^ MSC	Platelet lysate add	Centeno et al. [105]
	Knee OA	15	BM-MSC	40.9 ± 0.4 × 10^6^	–	Soler et al. [106]
	Knee OA	4	BM-MSC	8–9 × 10^6^	–	Davatchi et al. [107]
	Knee OA	4(3)	BM-MSC	8–9 × 10^6^	–	Davatchi et al. [108]
	Knee OA	24	BM-MSC	1.3 × 10^7^	Collagen gel	Wakitani et al. [109]
	Knee OA	56	BM-MSC	1.46 ± 0.29 × 10^7^	Hyalurone	Wong et al. [110]
	Knee OA	26	UC-MSC	20 × 10^6^ (1 vs. 2 times)	–	Matas et al. [111]

*BM-MSC* bone marrow-derived mesenchymal stromal cells, *AD-MSC* adipose tissue-derived mesenchymal stromal cells, *UC-MSC* umbilical cord-derived mesenchymal stromal cells, *BMAC* bone marrow aspirate concentrate, *ONFH* osteonecrosis of the femoral head, *OA* osteoarthritis
